# Image-guided percutaneous sclerotherapy of venous malformations of the head and neck: Clinical and MR-based volumetric mid-term outcome

**DOI:** 10.1371/journal.pone.0241347

**Published:** 2020-10-29

**Authors:** Dominik F. Vollherbst, Philipp Gebhart, Steffen Kargus, Astrid Burger, Reinald Kühle, Patrick Günther, Jürgen Hoffmann, Martin Bendszus, Markus A. Möhlenbruch

**Affiliations:** 1 Department of Neuroradiology, Heidelberg University Hospital, Heidelberg, Germany; 2 Department of Oral and Maxillofacial Surgery, Heidelberg University Hospital, Heidelberg, Germany; 3 Department of Pediatric Surgery, Heidelberg University Hospital, Heidelberg, Germany; Kepler University Hospital: Kepler Universitatsklinikum GmbH, AUSTRIA

## Abstract

**Objective:**

To report the clinical and MRI-based volumetric mid-term outcome after image guided percutaneous sclerotherapy (PS) of venous malformations (VM) of the head and neck.

**Methods:**

A retrospective analysis of a prospectively maintained database was performed, including patients with VM of the head and neck who were treated with PS. Only patients with available pre- and post-interventional MRI were included into this study. Clinical outcome, which was subjectively assessed by the patients, their parents (for paediatric patients) and/or the physicians, was categorized as worse, unchanged, minor or major improvement. Radiological outcome, determined by MRI-based volumetric measurements, was categorized as worse (>10% increase), unchanged (≤10% increase to <10% decrease), minor (≥10% to <25% decrease), intermediate (≥25% to <50% decrease) or major improvement (≥50% decrease).

**Results:**

Twenty-seven patients were treated in 51 treatment sessions. After a mean follow-up of 31 months, clinical outcome was worse for 7.4%, unchanged for 3.7% of the patients, while there was minor and major improvement for 7.4% and 81.5%, respectively. In the volumetric imaging analysis 7.4% of the VMs were worse and 14.8% were unchanged. Minor improvement was observed in 22.2%, intermediate improvement in 44.4% and major improvement in 11.1%. The rate of permanent complications was 3.7%.

**Conclusion:**

PS can be an effective therapy to treat the symptoms of patients with VMs of the head and neck and to downsize the VMs. MRI-based volumetry can be used to objectively follow the change in size of the VMs after PS. Relief of symptoms frequently does not require substantial volume reduction.

## Introduction

Venous malformations (VMs) are congenital slow-flow vascular malformations with a wide range in size and appearance [1, 2]. Diagnosis and treatment can be difficult, as there is a broad spectrum of symptoms, as well as of clinical and imaging findings [3–5]. Therefore, the International Society for the Study of Vascular Anomalies (ISSVA) developed a categorization schema based on the inventiveness of Mulliken and Glowacki [1, 6]. Cornerstones of this categorization are the differentiation between vascular tumours and vascular malformations and the subdivision of vascular malformations into simple malformations (e.g. venous malformations, lymphatic malformations and high-flow lesions, such as arteriovenous malformations and fistulas) and combined malformations (e.g. venolymphatic malformations). Due to the various manifestations of VMs, there are a lot of possible symptoms. Common symptoms include cosmetic disfigurement, pain and compression of adjacent anatomical structures. Percutaneous sclerotherapy (PS) is one of the most established treatment methods in combating those symptoms [7, 8]. The aim of this therapy is to induce sterile inflammatory reactions, involving the endothelium, which finally leads to occlusion of the affected vessels and involution of the vascular malformation [9, 10]. Diagnosis is usually based on the clinical history, clinical examination and magnetic resonance imaging (MRI) [11, 12]. Although MRI is a precise and established examination technique, a review of the literature discloses a lack of detailed volume measurement to evaluate the outcome after the treatment of VMs [4, 13–16]. The aim of this study was to report the clinical and MRI-based volumetric mid-term outcome in patients with venous malformations of the head and neck who were treated with percutaneous sclerotherapy in our tertiary care centre and to investigate the differences and correlation between clinical and radiological outcomes.

## Materials and methods

A retrospective analysis of a prospectively maintained patient database was performed in order to identify all subsequent patients with VMs of the head and neck region who were treated with image-guided percutaneous sclerotherapy from September 2010 to May 2015. Study approval and written informed consent for data collection were provided by the local ethics committee (ethical committee of the Medical Faculty Heidelberg, reference number: S-247/2009) and by the patients, respectively. Patient demographics, clinical presentation and clinical follow-up were assessed and recorded.

Patients with VM of the head and neck with a minimum follow up period of 12 months and with available pre- and post-interventional MRI were included into this study. Patients with mixed malformations, patients with follow up periods <12 months and patients without pre- and post-interventional MRI were excluded from this study.

### Treatment strategy

For the determination of the treatment strategy, each patient who suffered from a VM of the head and neck, was presented to an interdisciplinary conference, consisting of an interventional neuroradiologist, an oral and maxillofacial surgeon and, for pediatric patients, a pediatric surgeon. After interdisciplinary decision making, PS was offered to the patient. The aims of the therapy were both symptom mitigation and size reduction [11, 17].

The agents used for sclerotherapy were polidocanol (Kreussler & Co. GmbH, Wiesbaden, Germany) or ethanol 95% (B. Braun, Melsungen, Germany). The choice of the sclerosing agent depended on the number and success of previous treatments, on the size of the lesion and on the adjacent structures. Polidocanol was predominantly used as the first-line sclerosing agents. After one or more ineffective polidocanol treatments, highly-concentrated ethanol was combined with polidocanol or used instead, since ethanol is more effective, while it bears more complications. On the other hand, because of its more aggressive effect, ethanol was avoided in cases of vulnerable nearby structures.

### Treatment technique

PS was performed under sterile conditions, general anaesthesia and fluoroscopy guidance (Artis zee; Siemens, Forchheim, Germany). General anesthesia was performed to prevent discomfort, agitation and pain (which was observed several times in our institution during sclerotherapy under local anesthesia before the study period). Percutaneous puncture of the VMs was performed using 21 G or 23 G needles with a length of 19 mm, connected to a 200x2.3 mm plastic tube (Safety-Multifly; Sarstedt AG & Co. KG, Nümbrecht, Germany). After puncture using one or two needles, proper position was confirmed by spontaneous backflow of blood into the plastic tube and by performing a digital subtraction angiography of the VM using iodinated contrast agent (Solutrast 300, Bracco Imaging, Konstanz, Germany). According to the Tessari technique, in most of the cases, air and sclerosing agent were primarily compounded in a ratio, ranging from 1:2 to 1:1, applying a double-syringe-system-technique [18].

### Clinical evaluation criteria

The clinical follow-up examination was performed by both a neuroradiological physician and a surgeon. Clinical assessment included the visual aspect of the VM (with the aid of photo documentation), the subjective symptoms of the patients, and/or the impression of the parents and/or the physicians (the latter especially in children in whom anamnesis was impeded or not possible). In case of disagreement between patient’s/parent’s impression and the impression of the physicians, the impression of patients/parents was declared as the final outcome. Clinical outcome was categorized as 0 = worse, 1 = unchanged, 2 = minor improvement or 3 = major improvement.

### MRI

MRI was performed not more than 4 weeks before the first sclerotherapy treatment and 6 to 8 weeks after the last sclerotherapy session using a 3T MRI system (Magnetom Trio or Verio, Siemens, Erlangen, Germany) with a standard head coil applying standard non-enhanced and contrast-enhanced T1- (TR: 600 ms, TE: 12 ms) and T2-weighted (TR: 4240 ms, TE: 67 ms) turbo spin echo (TSE) sequences with a field of view of 230 mm and a slice thickness of 2.0 mm. Axial and coronal images were acquired. Measurements were performed on T2-weighted sequences (with or without fat saturation, depending on the location of the VMs), while T1-weighted sequences were used as an aid when measuring the volume was difficult. For pediatric patients MRI was performed with the patient under general anesthesia, if necessary.

### MRI-based volumetry

The assessment of the volume of the VMs was performed on pre-interventional MR images and on MR images after the last treatment session using a special workstation (Leonardo; Siemens, Erlangen, Germany). For the volume calculation, each lesion was manually delineated on each slice by means of manual segmentation. Most of the VMs were measured in the axial plane. Sagittal or coronal planes were used instead if they allowed a better delineation of the lesion.

Response to treatment was evaluated based on the calculated volume and categorized into: 0 = worse (>10% increase), 1 = unchanged (≤10% increase up to <10% decrease), 2 = minor improvement (≥10% decrease up to <25% decrease), 3 = intermediate improvement (≥25% decrease up to <50% decrease) or 4 = major improvement (≥50% decrease).

In order to investigate the effect of the size of the VM on the treatment outcome, the volumes of the treated VMs were divided into two groups based on the median volume. Consequently, VMs which were larger than the median were defined as “large VMs” and VMs which were smaller than the median were defined as “small VMs”.

### Adverse effects and complications

We differentiated between adverse effects, transient complications and permanent complications. Pain and swelling were regarded as adverse effects, as these reactions belong to the expected treatment effects after PS of VMs [19–22]. Adverse reactions others than pain and swelling were regarded as complications and categorized either as transient or permanent. Complications were categorized according to the CIRSE classification system for minimally-invasive techniques [23].

### Statistics

Prism (version 7.04; GraphPad, La Jolla, USA) was used for data analysis. To evaluate statistical differences between small and large VMs, the chi-square test for trend was performed. For statistical comparison of the difference between clinical and radiological outcome, radiological “intermediate improvement” and “minor improvement” were combined (resulting in 4-point ordinal scales respectively) and compared with the clinical “minor improvement” using the Wilcoxon matched-pairs signed rank test on each patient’s individual outcome. To assess the correlation between clinical outcome and absolute volume reduction, relative volume reduction and radiological outcome (5-point ordinal scale), respectively, the Spearman correlation coefficient was used. A p-value of 0.05 was considered as the threshold for statistical significance.

## Results

**Fig 1:**
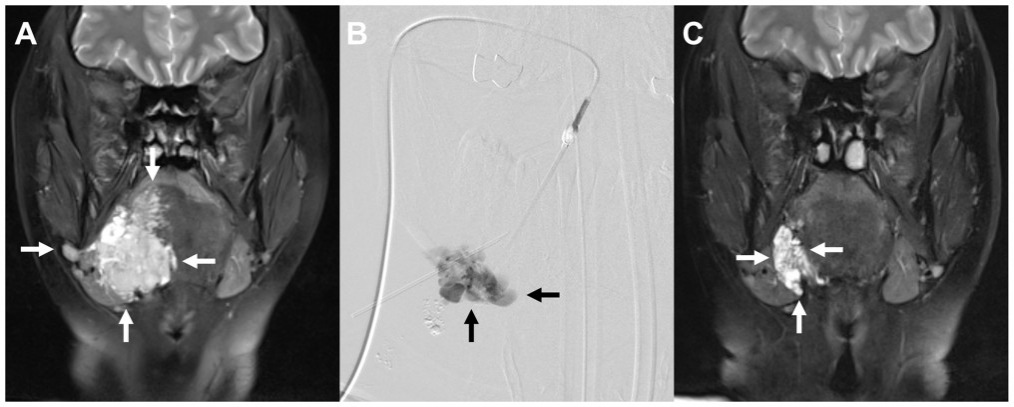
Illustration of an example case. This case demonstrates a 28-year-old female patient who suffered from pain and dysphagia, caused by a VM of the right-sided floor of mouth with involvement of the tongue. Pre-interventional T2w fat-saturated MRI shows the extent of the VM (white arrows in A). One percutaneous sclerotherapy session was performed under fluoroscopy guidance using 2 mL polidocanol 3% with foam. Intraprocedural digital subtraction angiography was performed to confirm the correct position of the needle (black arrows in B). In the MRI one year after the sclerotherapy (C) the size of the VM was significantly smaller (pre-interventional volume: 120.1 cm³, post-interventional volume: 65.4 cm³) and the patient reported substantial symptom relief.

Between September 2010 to May 2015, 27 patients (18 females) with a mean age of 29.2 years suffering from VMs of the head and neck were treated by PS. Each patient received between 1 and 3 sclerotherapy sessions (mean: 2.0). Overall, 51 sessions were performed. The mean time between following sclerosing sessions was 134 days (standard deviation (SD): 98 days), ranging from 49 to 443 days. Six patients (22.1%) were treated before their first sclerotherapy in our department. Four of them were treated by surgery only, one by laser therapy and one by both surgery and laser therapy. The procedural parameters are summarized in Table 1. One patient (3.7%) presented with an underlying syndrome (blue rubber bleb nevus syndrome). An example case if illustrated in Fig 1.

**Table 1:** Procedural parameters.

Sclerosing agent	Polidocanol 3% with foam	27 (52.9%)
*All treatment sessions*	Polidocanol 3% without foam	9 (17.6%)
	Polidocanol 2% with foam	1 (2.0%)
	Polidocanol 2% without foam	2 (3.9%)
	Polidocanol 1% with foam	1 (2.0%)
	Polidocanol 1% without foam	1 (2.0%)
	Ethanol 95% with polidocanol 3%	8 (15.7%)
	Ethanol 95%	2 (3.9%)
Sclerosing agent	Polidocanol 3% with foam	15 (55.5%)
*1st treatment session*	Polidocanol 3% without foam	6 (22.2%)
	Polidocanol 2% with foam	1 (3.7%)
	Polidocanol 2% without foam	1 (3.7%)
	Polidocanol 1% with foam	1 (3.7%)
	Polidocanol 1% without foam	1 (3.7)
	Ethanol 95% with polidocanol 3%	2 (7.4%)
Sclerosing agent	Polidocanol 3% with foam	9 (50.0%)
*2nd treatment session*	Polidocanol 3% without foam	3 (16.7%)
	Polidocanol 2% without foam	1 (5.6%)
	Ethanol 95% with polidocanol 3%	3 (16.7%)
	Ethanol 95%	2 (11.1%)
Sclerosing agent	Polidocanol 3% with foam	3 (50.0%)
*3rd treatment session*	Ethanol 95% with polidocanol 3%	3 (50.0%)
Volume of sclerosing agent (mL)^1^		6.0 ± 4.8 (1.0–18.0)
Treatment sessions	1	9 (33.3%)
	2	12 (44.4%)
	3	6 (22.2%)

Data are presented as No. (relative frequency in %) or mean ± SD (range)

^1^Total volume of injected sclerosing agent per session (without foam)

The most frequent treatment indication was pain in 12 cases (44.4%), followed by continuous growth in 11 cases (40.7%). Less common reasons for treatment were dysphagia in 4 cases (14.8%), cosmetic reasons in 3 cases (11.1%) and paraesthesia in 2 cases (7.4%).

### Clinical outcome

After a mean follow-up of 31 months (SD: 20.1 months; follow-up period beginning after the last treatment), the clinical outcome was categorized as worse for 2 patients (7.4%) and unchanged for 1 patient (3.7%). For 2 patients (7.4%) there was minor improvement, while there was major improvement for 22 patients (81.5%).

### Radiological outcome

Pre- and postinterventional MRI-based volumetry was available for all patients. The volume of the VMs ranged between 1.0 cm^3^ and 984.4 cm^3^ before the first PS session. The mean size was 117.3 cm^3^ (SD: 226.0 cm^3^).

Radiological outcome was worse for 2 lesions (7.4%) and unchanged for 4 lesions (14.8%), respectively. All but two VMs (92.6%) showed volume reduction on MRI. Six of these lesions (22.2%) showed minor improvement, 12 (44.4%) showed intermediate improvement and 3 (11.1%) showed major improvement.

### Differences between small and large VMs

**Fig 2:**
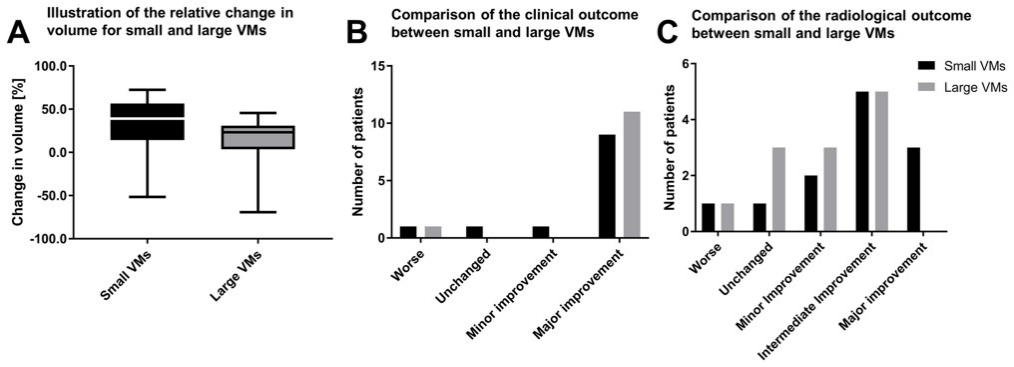
Comparison of small and large VMs. For smaller venous malformations, there was a broad spectrum of therapy response, ranging from a near cue up to a considerable increase in size (A). For larger venous malformations, the range in therapy response was comparatively smaller. Neither the clinical outcome (B), nor the radiological outcome (C) was significantly different between small and large VMs.

When comparing small and large VMs, there was a broader spectrum of radiological therapy response for smaller VMs, ranging from a near cure up to a considerable increase in size (Fig 2A). However, there was no statistically significant difference in the clinical (p = 0.493) or radiological (p = 0.153) outcome between small and large VMs (Fig 2B and 2C).

The clinical and radiological outcome is summarized in Table 2.

**Table 2:** Clinical and radiological outcome.

		All VMs	Small VMs	Large VMs
Volume		117.3 ± 207.3 cm³	19.9 ± 11.3 cm³	2020.3 ± 297.2 cm³
Range		1.0–984.4 cm³	1.0–39.0 cm³	45.8–984.4 cm³
Clinical outcome	Worse	2 (7.4%)	1 (7.7%)	1 (7.7%)
	Unchanged	1 (3.7%)	1 (7.7%)	0 (0%)
	Minor improvement	2 (7.4%)	1 (7.7%)	1 (7.7%)
	Major improvement	22 (81.4%)	10 (76.9%)	11 (84.6%)
Radiological outcome	Worse	2 (7.4%)	1 (7.7%)	1 (7.7%)
	Unchanged	4 (14.8%)	1 (7.7%)	3 (23.1%)
	Minor improvement	6 (22.2%)	2 (15.4%)	4 (30.8%)
	Intermediate improvement	12 (44.4%)	6 (46.2%)	5 (38.5%)
	Major improvement	3 (11.1%)	3 (23.1%)	0 (0%)

Data are presented as No. (relative frequency in %) or mean ± SD

### Difference and correlation between clinical and radiological outcome

**Fig 3:**
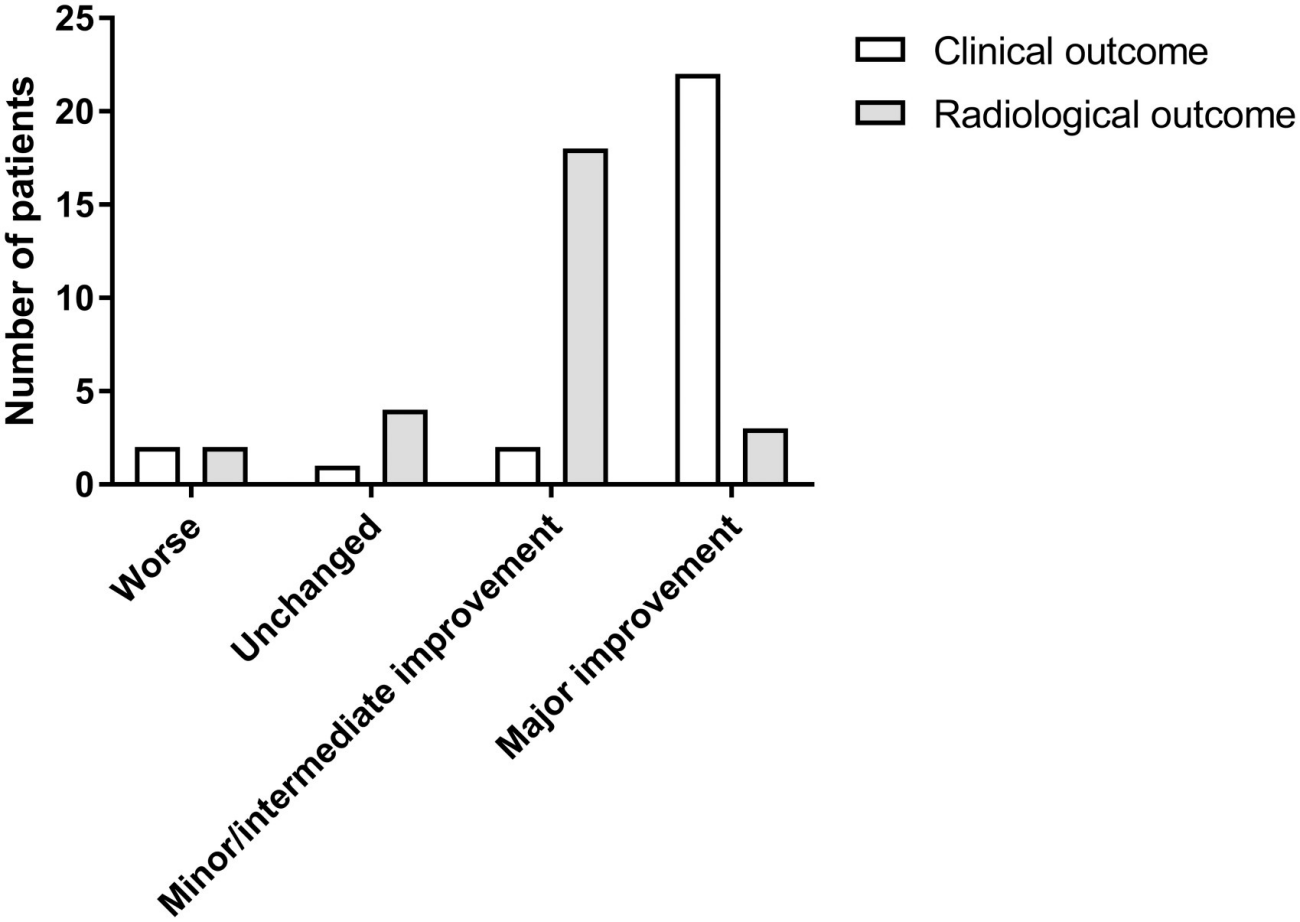
Comparison of clinical and radiological outcome. Statistical analysis showed a significant difference between clinical and radiological outcomes.

The difference between clinical and radiological outcome is illustrated in Fig 3. There was statistically significant difference between the clinical and radiological outcome (p<0.001). For example, there was major clinical improvement for 81.4 of the patients, while the frequency of major radiological improvement was only 11.1%.

The results of the correlation analysis are presented in Table 3. There was a statistically significant correlation between absolute volume reduction and clinical outcome (p = 0.003), while there was no significant correlation between clinical outcome and relative volume reduction (p = 0.423) or radiological outcome (p = 0.391), respectively.

**Table 3:** Correlation analysis.

	Clinical outcome vs. relative volume reduction	Clinical outcome vs. absolute volume reduction	Clinical outcome vs. radiological outcome
Spearman r	0.544	0.161	0.172
95% confidence interval	-0.1947–0.7703	-0.245–0.518	-0.234–0.527
p-value	0.003	0.423	0.391

### Adverse effects and complications

With regard to adverse effects after PS, swelling of the treated region was observed after 24 PS sessions (88.9%) and pain occurred after 17 treatment sessions (63.0%). Transient complications included haemorrhage in 2 cases (7.4%; complication grade 1), which could be stopped by manual compression, and temporary sinus bradycardia in 1 case (3.7%; complication grade 1) in which ethanol was used as the sclerosing agent for the treatment of a VM located at the base of the mouth. One permanent complication was observed (3.7%) which consisted of cicatrisation of the skin after two sclerotherapy sessions of a superficially located VM of the cheek using polidocanol 3% with foam (complication grade 4). The cicatrisation was substantially decreasing during the follow-up period 18 months and did not require any additional treatment.

## Discussion

PS is one of the therapy methods of choice for treating VMs since it is an effective minimally invasive technique to alleviate symptoms and to achieve reduction in volume.

Although PS is an established therapy method for the treatment of VMs of the head and neck region, an objective pre- and post-treatment evaluation by MRI has not been comprehensively reported in the literature [8, 11, 13, 14, 24]. In most studies, the only parameter for assessment of the size of the VM was the maximum diameter of the lesion [4, 13, 14]. Only in the study published by Alexander et al., investigating clinical and radiological treatment response after PS of lymphatic and VMs, volumetric analyses were performed [25]. To optimise the objective outcome measure after PS of VMs we determined the size of the lesions more precisely by using detailed volumetric analyses. Multiple MR-slices were analysed for each lesion of each patient and both the surface and the extent of the malformations were quantified in order to determine their volume. Using this detailed analysis, even for VMs with diffuse configuration, the volume was calculated.

When comparing clinical and MRI volumetry-based outcomes, there was a significant difference between the clinical and radiological outcome in our study. The correlation analysis showed a statistically significant correlation between clinical outcome and absolute volume reduction. However, there was no significant correlation between the clinical outcome and relative volume change or the radiological outcome (5-point ordinal scale). These findings demonstrate that clinical and radiological outcome after PS of VM often does not match and that even minor decreases in size can lead to a substantially better clinical outcome. A possible explanation for this finding is that in most cases, physicians and patients primarily assess the external appearance of the lesion, making the superficial components, which are mostly the first target of PS, to account more for the clinical outcome, while deeper structures often constitute the major part of the lesion’s volume. Furthermore, the superficial compartments of VMs are often the primary reason for symptoms, predominantly for pain [1]. PS of these superficial compartments often caused symptom remission while the volume of the lesion was only slightly decreased.

We divided the collective of head and neck VMs into two groups of smaller and larger lesions, based on the median of the initial volume of the VMs. It became apparent that small VMs had a broad spectrum ranging from a near cure up to a considerable increase in size, whereas the bigger VMs had a smaller range of treatment response. However, there was no significant difference in clinical or radiological outcome when comparing small and large VMs.

Several studies investigated the efficacy of PS regarding the patient’s symptoms and the volume change of the VMs. In a recent study published Khaitovich et al. including 309 patients with VMs throughout the whole body, complete symptom relief ranged between 12% and 23% (depending on the type of symptom) [8]. Specific MRI-based lesion analyses, however, were not performed. Spence et al. included 37 patients in their study treating facial VMs with bleomycin [14]. In this study, in which the lesions were measured in their maximum diameter, 21 of 32 lesions (65.6%) showed objective improvement on MR imaging. Furthermore, Spence et al. compared their objective results with subjective clinical results. Subjectively, 29 of 32 patients (90.6%) noticed improvement and in 30 cases (93.8%) the clinicians classified the lesions as improved [14]. Yun et al. performed a retrospective study of 158 VM patients. In 123 cases, the objective evaluation was based on the maximal diameter of the VMs on MR imaging. 22 lesions (17.9%) showed an increase of 10% or more and 57 lesions (46.3%) no or minor change (<10%). Yun et al. based their subjective clinical outcomes on a self-assess questionnaire and differed between symptomatic, cosmetic and functional improvement. Regarding the symptomatic outcome they found out that 26 of 94 patients (27.7%) reported a marked improvement and 19 patients (20.1%) reported a moderate improvement. Almost half of the patients (46.8%) did not perceive any change. 5 Patients (5.3%) reported “moderately worse” or “markedly worse” [26]. In our study, a comparatively lower number of treatment cases showed substantial volume reduction on MRI. However, objective evaluation in our study was different from the studies mentioned above, as we calculated the volume of each lesion. Furthermore, we analysed the change in volume of the VMs with reference to their initial volume.

We distinguished between adverse effects and complications since some adverse effects belong to the expected physiological reaction after PS of VMs. However, the seamless transition between adverse effects and complications made it difficult exactly to distinguish these categories in some cases, which can be regarded as a drawback of this classification.

The rate of adverse effects and of complications in our study is comparable to the literature. Mimura et al. reported that transient local swelling occurred in 50 of 59 sclerosing sessions (84.7%) and transient local numbness 3 times (5.1%) [22]. Spence et al. compared alcohol and bleomycin sclerotherapy [4]. 23.5% of the patients who were treated with alcohol developed nerve palsies, which resolved completely in 3 patients, while 11.8% of the patients suffered from transient severe local swelling.

The most commonly used sclerosing agents are polidocanol, sotradecol, ethanol, sodium morrhuate and pingyangmycin [27]. We decided to use polidocanol as the primary sclerosing agent since polidocanol is less aggressive than ethanol with a comparatively lower risk of complications. Another reason for using polidocanol was the location of the VMs. Therefore, we avoided the use of ethanol as far as possible, due to its aggressive sclerosing effect in order to minimize the risk of complications, which can be dangerous especially in the head and neck region [22, 28]. In a study by Berenger et al. treating patients with ethanol or sodium tetradecyl sulphate, major complications included ulceration and scarring (13%), acute blistering (50%) and transient facial paresis (5%) [29]. Orlando et al. followed 81 patients after ethanol sclerotherapy. 13.6% of them developed skin ulcer, 3.7% hyperpigmentation and 3.7% paraesthesia [19].

This study was conducted in a single institution with retrospective analysis of prospectively-maintained data, which can be regarded as a potential limitation on the study findings. Another potential limitation is a selection bias caused by the indication for MRI, since patients who were free of symptoms after PS did not receive an MRI after treatment. This bias could have led to an underestimation of the treatment success with regard to the volumetric analysis.

## Conclusions

PS of VMs of the head and neck region is a safe technique to reduce symptoms and achieve substantial volume reduction. MRI-based volumetry can be used to objectively follow the change in size of the VMs after PS. Symptom relief does often not require substantial objective volume reduction.

## Supporting information

S1 Table Dataset of the study’s results. (XLSX)
